# Potential Role of Sleep Deficiency in Inducing Immune Dysfunction

**DOI:** 10.3390/biomedicines10092159

**Published:** 2022-09-01

**Authors:** Kasper Kuna, Krzysztof Szewczyk, Agata Gabryelska, Piotr Białasiewicz, Marta Ditmer, Dominik Strzelecki, Marcin Sochal

**Affiliations:** 1 Department of Sleep Medicine and Metabolic Disorders, Medical University of Lodz, 92-215 Lodz, Poland; 2 Department of Affective and Psychotic Disorders, Medical University of Lodz, 92-213 Lodz, Poland

**Keywords:** immune system, vaccination, autoantibodies, psychoneuroimmunology, sleep deficiency, insomnia

## Abstract

Sleep deficiency and insomnia deteriorate the quality of patients’ lives, yet the exact influence of these factors on the immune system has only begun to gain interest in recent years. Growing evidence shows that insomnia is a risk factor for numerous diseases, including common infections and autoimmune diseases. Levels of inflammatory markers also seem to be abnormal in sleep deficient individuals, which may lead to low-grade inflammation. The interpretation of studies is difficult due to the equivocal term “sleep disturbances,” as well as due to the various criteria used in studies. This narrative review aims to summarize the available knowledge regarding the bidirectional influence of the immune system and sleep disturbances.

## 1. Introduction

A considerable number of studies focused on insomnia and sleep deficiency have suggested that chronic forms of those conditions are risk factors for arterial hypertension [1,2], diabetes mellitus [3], obesity, depression [4,5], or Alzheimer’s disease [6,7]. Growing evidence associates sleep deficiency with immune alteration, which can lead to lower vaccination efficiency [8] as well as a higher risk of autoimmune diseases [9]. Furthermore, those conditions were also identified as risk factors for other immune system dysfunctions such as rheumatoid arthritis (RA) [9], inflammatory bowel disease [10,11], or Hashimoto’s disease [9,12]. The term immunity could be defined as the entirety of immune reactions to microbes, molecules recognized as intruders, noninfectious molecules, and products of our damaged cells or tumor cells [13]. 

The central nervous and immune systems work closely in responding to external stimuli and pathogens. This interplay occurs by several mechanisms. Sympathetic, peptidergic, and sensory nerve fibers reach primary and secondary lymphoid organs. Neuropeptides secreted by the neuronal endings in tissues can be detected by macrophages [14], thus inducing immune reaction. Another way of transferring information from the central nervous system (CNS) to the immune system is by neuroendocrine mediators secreted by the hypothalamus, pituitary gland, and adrenal glands, which leukocytes can sense. Those cells can also synthesize the aforementioned mediators resulting in the paracrine effect [14,15,16]. All those mechanisms enable communication between the immune system and the CNS, inducing fever or sleepiness. Most of the studies, based on animal models, point to IL-1 and TNF-α as the main sleep-immune regulators. In general, pro-inflammatory cytokines increase the duration of NREM sleep, while anti-inflammatory agents have the opposite effect in animal models [17]. Human studies concerning this topic are scarce. However, similar mediators were shown to substantially affect sleep, particularly IL-1, TNF-α, and prostaglandins [17]. All those mechanisms enable communication between the immune system and the CNS, inducing fever or sleepiness. Interestingly, papers concerning inflammatory diseases such as Crohn’s disease or RA show that anti-TNF-α therapy may improve sleep quality, independent of alleviating the severity of the disease and pain [18,19]. One example is a report by Penner et al., which examined Natalizumab, a monoclonal antibody that binds to α4β1 integrin on white blood cells. In effect, drugs stop those cells from crossing the blood–brain barrier, resulting in reduced inflammation. Results showed that multiple sclerosis patients who received natalizumab had improved daytime sleepiness [20].

The difficulties in the research of immune dysfunction concurrent with sleep deprivation are caused mainly by the variety of adopted models of insomnia and sleep deprivation. Some studies use only one-night sleep deprivation, whereas others observe alterations during sleep deprivation, which lasts for several days. Similarly, not all studies concerning insomnia used the same diagnostic criteria, which resulted in discrepancies in the inclusion to the study group. There are also different insomnia phenotypes; the two most commonly researched are insomnia with short sleep duration (usually sleep duration < 6.5 h per night) and insomnia with normal sleep duration (usually sleep duration > 6.5 h per night) [1,21,22]. What is important is that most research papers do not account for this distinction. Additionally, there is self-inflicted short sleep duration and sleep deprivation associated with other conditions such as obstructive sleep apnea or restless leg syndrome. Given differentiated inclusion criteria and the diversity of the illnesses, data on the influence of chronic limited sleep time on the immune system are somewhat ambiguous and often inconsistent [23]. 

The PubMed (https://pubmed.ncbi.nlm.nih.gov, accessed on 25 May 2022), Google Scholar (https://scholar.google.com, accessed on 25 May 2022), and Scopus (https://www.scopus.com, accessed on 25 May 2022) databases were searched with the following keywords (sleep OR insomnia OR sleep deprivation OR sleep deficiency) AND (immunity OR vaccination efficacy OR cytokines OR inflammation OR autoimmune diseases OR cortisol). The search was limited to publications in the English language published up to 31 December 2021, with updates on 18 August 2022. Relevant studies were analyzed, and disagreements were resolved by discussion. 

In this narrative review, we summarize knowledge about the influence of chronic sleep deficiency on immune system functioning. Furthermore, we discuss altered inflammatory responses in patients suffering from insomnia or other forms of sleep deficiency. Our report also concerns the risks of various immune-related diseases, along with impaired vaccination efficiency in the course of insomnia and the presence of sleep deficiency. We specify, whenever possible, which sleep disorders the article is concerned with, and adopt the terminology used by its authors (insomnia, sleep deprivation, or short sleep time).

## 2. The Interrelation between Sleep and Alterations in Inflammatory Markers Secretion

The relation between sleep and immunity is bidirectional, since inflammation can cause sleepiness, but also in some pathological conditions such as prolonged inflammatory response, it can also disrupt circadian rhythm [17]. Most studies dealing with alterations in inflammatory markers focused on acute sleep deprivation or restriction. There have been only a few studies on the effects of long sleep deprivation and habitual sleep loss on the various inflammatory markers.

### 2.1. Inflammatory Markers in Sleep Deprivation Model: The Insight into Animal Studies

Proinflammatory cytokines are known to increase sleepiness. Intraventricular administration of IL-1, tumor necrosis factor-α (TNF-α), or IFN-α in rabbits and rats induces a 60 to 70% increase of non-rapid eye movement (NREM) sleep duration [24]. A study performed on sleep-deprived rats showed increased TNF-α and IL-6 levels, as well as expression of phosphorylated nuclear factor kappa-light-chain-enhancer of activated B cells (NF-κB) in retroperitoneal adipose tissue [25]. Another study on rats showed elevated proinflammatory cytokines levels (especially IL-1-α, IL-1β, and IL-6) in rats subjected to 72 h of rapid eye movement (REM) sleep deprivation. Seven days after the experiment, IL-1α, IL-1β, and IL-6 levels were unremarkable. However, levels of IL-17A and TNF-α remained elevated in the experimental group [26]. These results suggest that some inflammatory effects of sleep deprivation may be long-lasting and possibly are not easily reversible, thus impacting the risk of autoimmune diseases.

Baracchi et al. [27] investigated differences in sleep parameters between groups of mice with a hereditary lack of both IL-1βR1 and TNF-αR1 and normal subjects. During the experiment, mice were exposed to 12 h of light and dark cycles. The experimental group was found to spend less time in NREM during the period of darkness, and in a 24-h cycle, less time in REM. It was shown that after 6 h of sleep deprivation, the experimental group did not exhibit rebound REM sleep, contrary to the control group. The increase of NREM sleep was present only in the first 6 h after sleep deprivation in the experimental group and was lower than in the control group [27]. These outcomes indicate bidirectional interactions between the immune system and sleep.

Another study on mice concerning lymphotoxin, a member of the TNF- superfamily of cytokines, also known as TNF-β, examined differences between four groups: those lacking both TNF-α and lymphotoxin-α (Ligand Knock-out (KO)), lacking TNF-α receptors 1 (Receptor 1 KO), lacking TNF-α receptors 2, and the control group. During the experiment, mice were exposed to 12 h of light and dark cycles. There was no difference in general sleep duration between all groups. However, the duration of REM sleep was decreased in Receptor-1 KO and Ligand KO mice. This phenomenon was restricted only to the light period. However, in dark periods all KO mice exhibited increased REM sleep duration but with lesser frequency. Surprisingly, no significant differences were observed between the groups after sleep deprivation [28]. This study adopts a unique perspective and may indicate that inflammatory markers mainly affect REM sleep.

Studies on animal models indicate that IL-1 and TNF may play a role in sleep onset and retention. In animal models of mice, some inflammatory effects of sleep loss are not easily reversed after sleep normalization. Additionally, it emerges that not only does inflammation influences sleep quality, but also sleep disturbances have a fundamental impact on inflammatory mediators’ levels and activity. 

### 2.2. Interaction between Inflammation and Sleep Disturbances—Human Studies 

Sleep deprivation promotes an increase of various inflammatory markers. However, mediators such as IL-6, TNF-α, and CRP [22,29,30] appear to be the most significant in human studies. Furthermore, both IL-1β and TNF-α bind to their activating receptors on neurons, causing altered neuronal excitability and function. Through this signaling pathway, they promote NREM sleep and can lead to sleepiness, fatigue, and poor cognition [31,32]. However, results in this field are often inconsistent, mainly caused by the heterogeneity of adopted definitions. Part of the trials investigate patients suffering from insomnia, while other researchers focus on sleep disturbances or short sleep duration. 

Insomnia is defined as difficulties initiating or maintaining sleep and poor sleep quality, which disturbs everyday activities. According to the International Classification of Diseases (ICD), insomnia could be as frequent as 15%, while using the 5th edition of the Diagnostic and Statistical Manual of Mental Disorders (DSM-V), the prevalence of insomnia is estimated at 11% [33]. The biological basis of insomnia is the internal dysfunction of mechanisms responsible for the sleep–wake cycle. On the other hand, sleep deprivation is an example of sleep disturbance, which does not fulfill diagnostic criteria for insomnia since it is caused by external factors, independent from the patient, e.g., noise or internal stimuli such as pain.

Chronic insomnia patients were found to have an increased level of IL-6 during the second half of the night. Moreover, subjective sleep duration and quality were suggested to correlate negatively with IL-6 levels [29]. In another study on insomnia, symptoms with objective short sleep duration (<7 h) were associated with elevated levels of CRP in adolescents compared to the other groups, including those with objective short sleep duration without insomnia symptoms [22]. This is consistent with other research, in which insomnia symptoms were associated with higher CRP levels in young adults (21–35 years old). Interestingly, this association remained independent and significant when neuroticism and depression were included in the model [34], which highly implies that objective short sleep duration with insomnia symptoms directly correlates with CRP elevation. On the other hand, in a study by Prather et al., patients with longer self-reported sleep duration (>10 h) had significantly higher levels of CRP and IL-6. It is worth noting that the Insomnia rating scale scores were not correlated with serum levels of these cytokines [35]. 

A meta-analysis assessing more than 50,000 patients revealed a significant increase of CRP and IL-6 levels in subjects suffering from sleep disturbances (confirmed by questionnaires, interviews, or insomnia diagnosis). Surprisingly, this result was not observed for the group clinically diagnosed with insomnia on its own. As for studies regarding experimental sleep deprivation included in this meta-analysis, there was no impact on CRP, IL-6, or TNF-α levels. Individual studies showed that women were more vulnerable to sleep disturbance, with a higher increase of CRP, IL-6, and NF-κB. Authors pointed out that associations between sleep duration and inflammation are similar to those of sleep and mortality, in which long and short sleepers have a greater risk of dying than normal sleepers [23], which is consistent with another study that states insomnia with an objective shorter sleep duration is connected with increased morbidity and mortality [36]. Directions of associations between insomnia and pro-inflammatory cytokines are elusive, as none of the included studies were designed prospectively.

A report by Watson et al. adopted a rare method, recruiting eleven pairs of monozygotic twins with diverged sleep time compared to their siblings. Results showed that peripheral blood leukocytes in monozygotic twins with shorter sleep than their siblings had increased expressions of genes associated with ribosomal, transcriptional, and translational processes. Worth noting that those discrepancies were minor, with most genes changing by less than 2-fold. Surprisingly, peripheral blood leukocytes in this group also had down-regulated genes associated with interleukin signaling pathways (Il-2, IL-4, Il-6, and IL-8), interferon signaling, phagocytosis, and granulocyte-macrophage colony-stimulating factor signaling, as well as pro-inflammatory messengers, namely the Janus kinase–signal transducer and activator of transcription system [37]. There were also several overlapping pathways with other studies, e.g., suppression of pathways involved in natural killer cell signaling. This report indicates that immune reactions for habitual sleep curtailment may differ from those observed in a short sleep restriction. Thus, more studies using, e.g., actigraphy or other methods of long-term sleep monitoring are granted.

Most studies associated sleep loss with an increase of markers such as IL-6, CRP, or TNF-α. This effect would partly explain the increased prevalence of inflammation-associated diseases such as diabetes mellitus (DM) or arterial hypertension (HA) in patients with sleep disturbances. However, a report by Watson et al. showed down-regulation of genes associated with interleukin signaling pathways in peripheral blood leukocytes of twins with habitual short sleep duration. Acquired methodology in this field is vastly diverging. Thus, unifying verbiage and measures in future research would allow for a better understanding of those processes.

### 2.3. Interaction of Sleep Medications with Inflammatory Mediators

Sleep medications used in the therapies of sleep disorders can also affect immune system functions. A study on mice showed that quetiapine increased serum levels of IL-10 and decreased IFN-γ 4 h after intraperitoneal injection of lipopolysaccharide, with similar results observed within the brain [38]. Unmetabolized quetiapine was also shown to increase levels of oxidative molecules (nitric oxide), protein levels, and proinflammatory cytokines (IL-α, IL-6, and TNF-α) in the in vitro setting [39].

Furthermore, pretreatment with trazodone inhibited inflammation-induced production of IL-6 and IFN-γ in TNF-α stimulated neuronal cells. Moreover, some TNF-α effects, including a decrease of neurotrophic and transcription factors (e.g., Brain-Derived Neurotrophic Factor, or cAMP response element-binding protein), and the production of inflammatory mediators, were counteracted by this drug. Trazodone alone was shown to decrease the release of IFN-γ [40]. Another sleep medication agent—melatonin, was shown to act as an antioxidative agent, NO-synthase inhibitor, downregulator of cyclooxygenase-2, and to facilitate numerous other mechanisms, which is of considerable interest for research of therapies for various diseases such as sepsis, neurodegenerative diseases, and sleep-related conditions [41,42]. However, melatonin also has some pro-inflammatory properties, such as releasing IL-β, IL-2, IL-6, IL-12, TNF-α, and IFN-γ in monocytes, monocyte-derived cells, and T-helper cells [42]. Since sleep-immune relation is bidirectional, the aforementioned sleep medications could also influence sleep via inflammatory pathways. However, those effects are not fully understood. Studies on monoclonal antibodies may provide some hints concerning this phenomenon.

It is established that high TNF-α levels induce sleep while low levels decrease sleepiness. This factor is mainly associated with sleep disturbances in patients suffering from conditions characterized by elevated TNF [43]. Despite unclear results of studies on TNF-α levels in humans with sleep disturbances, a study performed by Weinberger et al. showed that pharmacological (infliximab) inhibition of TNF-α improved sleep continuity, as well duration of total sleep time and stage 2 in depressed patients with inflammation (CRP > 5 mg/L), who did not respond to antidepressants and did not suffer from any other diseases. Lower soluble TNF-αR1 level was also correlated with decreased wake time after sleep onset and increased sleep efficiency in patients administered with infliximab, but only in participants with high baseline inflammation [44]. However, in RA patients with sleep disturbances (assessed with questionnaires), the anti-TNF-α therapy did not ameliorate sleep disturbances [45]. In a different study, patients with RA had increased median percentage sleep efficiency and decreased sleep latency after the first dose of infliximab (3 mg/kg) [19]. Notably, only six participants were recruited, one of whom suffered from obstructive sleep apnea. Another study showed improved Pittsburgh sleep quality index (PSQI) scores, but not polysomnography (PSG) parameters, in patients with active ankylosing spondylitis undergoing anti-TNF treatment [46]. There is also a report that tocilizumab therapy improved sleep quality in RA patients independently from disease activity [47]. 

This area of research demonstrates interesting preliminary results for sleep disturbances therapies. Sleep medications, besides their standard activity, may also influence cytokine levels in sleep-deprived patients. On the other hand, monoclonal antibodies, which target inflammatory mediators, appear to affect sleep quality. Those two processes deliver an illustrative showcase of the bidirectional relationship between sleep and the immune system. Additionally, sleep medications may alleviate risks connected with prolonged low-grade inflammation by counteracting the upregulation of inflammatory mediators. Nevertheless, more trials examining monoclonal antibodies and other sleep medications are needed to understand their impact on sleep.

### 2.4. Cell Mediated-Immunity Alterations in Sleep Deprivation or Natural Sleep Loss Observed in Sleep-Deprived Individuals

There is a report of lower CD3+, CD4+, and CD8+ cell count in insomniacs (diagnosis based on DSM-IV criteria) compared to healthy subjects [48]. Moreover, growth hormone, the maximum release of which is observed during slow wave sleep, was shown to promote the production of IFN-γ, which can contribute to the shift towards type 1 (IL-2, IL-12, IFN-g) activity of the T cells [49]. One study investigated the influence of natural short sleep on immune system functioning instead of experimental sleep deprivation. The group with natural short sleep (<7 h) had higher phytohemagglutinin-induced (T cell mitogen) T cell function and lower NK cells compared with normal sleepers (7–9 h) prior to immunization [50]. Phytohemagglutinin is a lectin and T cell mitogen. Therefore, this study suggests that short sleep duration may directly affect T cells, resulting in deteriorated cell-mediated immunity. In another study, participants were exposed to 5 days of sleep restriction (4 h in duration) and 7 days of sleep recovery (8 h in duration). During the days of sleep restriction, a gradual increase of white blood cells, monocyte, neutrophil, and lymphocyte count was observed. Although in the recovery phase, levels of monocytes and lymphocytes were decreasing, the level of neutrophils remained elevated at the end of the 7-day recovery period [51]. 

According to these results, the influence of sleep deficiency on cell-mediated immunity should be acknowledged. The activity of T cells appears to be dysregulated in sleep-deprived individuals, which may be associated with the deterioration of immune memory formation. Additionally, given the role of type 1 immunity in the pathophysiology of various autoimmune diseases, the impact of sleep on this type of immunity may partly explain the higher occurrence of autoimmune diseases in patients suffering from sleep disorders. Considering the limited number of studies investigating alterations in cell-mediated immunity caused by sleep deficiency, this interplay could not be fully recognized. Further studies and evidence are needed to corroborate or disprove this theory.

### 2.5. Summary

Sleep loss or deficiency influences the duration and quality of particular sleep phases. Research on the interaction between the immune system and insomnia may reveal new therapeutic directions (Table 1). Some authors noticed that administering monoclonal antibodies targeting inflammatory mediators impacts not only the symptoms of the primary disease, but also improves sleep quality. However, this could be caused by the alleviation of the symptoms of the disease. Nevertheless, it has been demonstrated that those therapeutic agents influence sleep parameters, regardless of the severity of the primary disease. However, those trials were conducted on small cohorts and acquired different protocols, preventing a definitive conclusion. Therefore, future studies should pay attention to accurate diagnostics of sleep disorders.

## 3. Sleep Deficiency and Its Influence on Autoimmunity

As has already been mentioned in this review, sleep appears to have a significant impact on immune functions; the results of many epidemiological studies show that impaired sleep increases the risk of autoimmune and chronic immune-related disease [9,12,52,53].

As Hsiao et al. show, non-sleep apnea sleep disorders increase the risk of systemic lupus erythematosus (SLE), RA, ankylosing spondylitis, and Sjögren’s syndrome (SS), with adjusted hazard ratios ranging from 1.45 to 1.81 [9]. Another prospective study in Norway on patients with insomnia revealed that they have an increased risk of developing RA within 11 years of the follow-up (Odds Ratio (OR): 1.87) [12]. Retrospective analysis of the Korean cohort focusing on the association between sleep disorders yielded results consistent with previous ones: subjects with sleep disorders had an increased risk of developing autoimmune conditions such as alopecia areata (OR 1.913), vitiligo (OR 1.539), Graves’ disease (OR: 1.717) and Hashimoto’s disease (OR: 1.641) [52]. Furthermore, in a study performed on relatives of patients with SLE, the incidence of developing this disease was higher in individuals who reported having <7 h of sleep per night (OR: 2.8) within the follow-up period of 6.3 (± 3.9) years [53].

The effects that disrupted sleep exert on the immune system are still underinvestigated. Sleep deprivation can accelerate the production of antinuclear antibodies (ANA) in mice (10–13 weeks of age vs. 13–16, sleep deprivation took place at the 11th week) and subsequent onset of SLE [54]. ANAs are also associated with other diseases, such as SS, scleroderma, polymyositis, and autoimmune hepatitis. As one case-control study showed, poor sleep was reported by 87.5% of multiple sclerosis patients in acute exacerbation (PSQI questionnaires assessing sleep over 1 month were taken within 7 days of the exacerbation) compared to 50% of those in remission [55]. Those results suggest that compromised quality or inadequate sleep duration might contribute to the onset of immune-mediated conditions or increase the risk of relapse. Thus, a vicious circle between ever-declining sleep quality and higher disease activity ensues [56,57,58,59].

In autoimmune or chronic inflammatory conditions, sleep disorders might be ascribed to mood disorders, anxiety, stress, etc., secondary to the underlying disease. Such a relation can be observed in e.g., MS. It is estimated that as much as 30.5% and 22.1% of MS patients suffer from depression or anxiety, respectively [60]. The prevalence of chronic insomnia disorder is similar; Viana et al. have shown that 22.3% of MS patients meet the criteria for diagnosis of chronic insomnia disorders according to the International Classification of Sleep Disorders (ICSD-3) [61]. Other sleep problems are even more common; Bamer et al. have surmised that even one out of every two MS patients could suffer from sleep problems [62]. Disease symptoms themselves might preclude afflicted individuals from having normal sleep; e.g., in male MS and female RA patients, the main determinant of sleep quality was pain [63]. Moreover, in this group, symptoms such as spasms and crawling feeling in the legs might be difficult to differentiate from restless leg syndrome, another sleep disorder frequently met in this population [63]. In IBD, nocturnal diarrhea might result in sleep fragmentation [64].

One theory suggests that sleep loss may result in an overload of reactive oxygen species (ROS) [65]. The exact reasons for ROS overload are not completely clear. It might be a byproduct of increased metabolism accompanying sleep disruptions or a result of endoplasmic reticulum stress potentially caused by an increase of misfolded/unfolded proteins or induced directly by sleep loss [65]. This accumulation can cause oxidative stress. Cell damage caused by oxidative stress might result in increased exposure to antigens that aren’t physiologically available to the immune system. This increase of antigen load might result in the production of autoantibodies [66]. In individuals with SLE, ANA and anti-double-stranded DNA (anti-dsDNA) antibodies were associated with advanced oxidation protein products [66]. Other authors report an association between anti-dsDNA antibodies and lipid peroxidation [66]. Since elevated production of autoantibodies might lead to the onset of immune-related diseases, sleep loss appears to be involved in the pathogenesis of such disorders.

Sleep disruptions might also be associated with impaired antioxidant defense, another potential cause for ROS overload. Night shift workers show weakened enzymatic (catalase, superoxide dismutase) and non-enzymatic (ferric reducing power) antioxidant defense, as well as higher levels of hydrogen peroxide and markers of plasma protein oxidation [67]. Melatonin, which has antioxidative properties, likely enhances this effect, since its secretion might be suppressed due to constant artificial light exposure at night [67]. Shift work might also be related to dysregulation of the HPA axis and associated alterations in cortisol secretion, which might affect DNA methylation and glutathione levels [63,67]. Since lower levels of catalase and glutathione peroxidase expressions are also associated with plasmocyte differentiation, it could be suspected that such conditions promote the production of autoantibodies and the development of autoimmune diseases [68].

**Figure 1:**
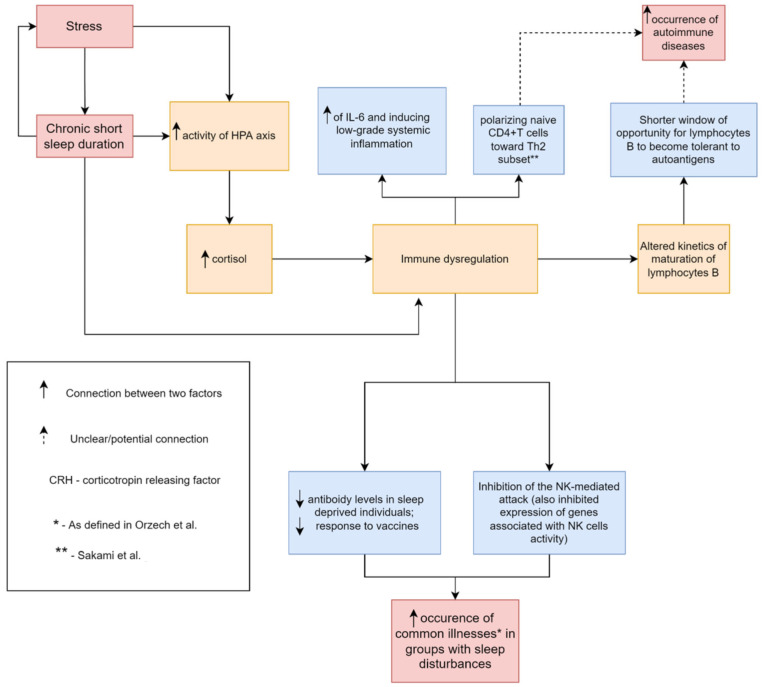
A common mechanism, by which sleep loss can contribute to increased occurrence of common illnesses such as cold, flu-like, gastroenteritis, menstrual pain, and increased occurrence of autoimmune diseases. Parts of the figure were drawn by using pictures from Servier Medical Art (http://smart.servier.com/), free and opened software, which is licensed under a Creative Commons Attribution 3.0 Unported License (https://creativecommons.org/licenses/by/3.0/). The picture was designed using diagrams.net website (Diagrams.net version 14.6.13, https://www.diagrams.net/, accessed on 18 June 2022).

As we mentioned earlier in this review, sleep deprivation is followed by the shifting of a cytokine profile to a more pro-inflammatory state. This might be explained by changes in the circadian clock, leading to NF-κB activation [69]. Increased levels of cortisol and catecholamines can contribute as well [70]. Those alterations might contribute to autoimmune processes. Hsiao et al. have also proposed that an increase of IL-17 and distorted regulatory T cells function might result in elevated autoantibody production [9]. Increased IL-6 levels can cause polyclonal B cell activation, which might promote autoantibody production [54] (Figure 1).

It seems that sleep disorders might have a deleterious effect on the course of chronic immune-mediated conditions. As one study shows, Crohn’s disease patients in remission with sleep disturbances (Patient-Reported Outcomes Measurement Information System T-score > 50) have a higher risk of relapse at 6 months compared to patients in remission without disturbed sleep [71]. Those effects can be attributed to the elevated autoantibody production promoted by changes in cytokine levels and oxidative stress. However, more studies on the subject are desirable.

In summary, sleep has a complex relationship with the immune system. Sleep disorders might impair mechanisms protecting against the development of autoaggression (e.g., antioxidant defense) and induce the production of pro-inflammatory cytokines, thus contributing to the production of autoantibodies (Table 2). However, due to a relatively low number and high variability in studies’ results as well as applied methods, mechanisms behind the influence of sleep deficiency on immune functions remain unclear. More research on this subject would contribute to a better understanding of the interactions between sleep and immunity.

## 4. May Sleep Deficiency Affect Vaccination Effectiveness? 

Prolonged sleep deprivation was suggested to decrease the immune response and memory formation, decrease vaccination efficiency, and increase the risk of infectious diseases, as well as deteriorate the course of some of them [72,73,74,75].

### 4.1. Vaccination Studies

The valuable models for determining the influence of sleep on the process of forming immunological memory are the vaccination studies, e.g., a study by Lange and colleagues showed that in the one-year follow-up, hepatitis A virus-specific Th cells were doubled in the group comprised of individuals, who had the opportunity to sleep 7.5 h compared to the group that stayed awake during the night following vaccination [76]. One of the mechanisms of the observed phenomena may be CD4+ T cell formation during sleep [77]. Similarly, in another research paper, there was a decreased immune response to hepatitis B vaccination in a group that slept less than 6 h. This study measured sleep with actigraphy 3 nights before and 3 nights after the immunization. Interestingly, discrepancies in anti-Hbs IgG levels were present 6-months following the third hepatitis B immunization [72]. Notably, sleep duration was associated with the decreased probability of clinical protection in the 6 months follow-up. Results of those studies are fragmentary, and investigations of the impact of habitual sleep loss on antibody production and vaccine efficiency are needed since this problem affects up to 35% of the United States adult population [78].

A different perspective of this interplay was examined in a study by Taylor et al. [8], which showed that insomnia might be a risk factor for decreased response to the influenza vaccine. Participants underwent a rigorous inclusion process consisting of completing the Insomnia severity index, Epworth sleepiness scale, PSQI, and Morningness/Eveningness questionnaire, as well as structured clinical interviews to rule out other mental or sleep disorders. The insomnia group had lower serum influenza antibody levels in one-year follow-up [8]. Similarly, more recent reports showed the association between shorter self-reported sleep duration and decreased antibody responses to influenza vaccination [79].

Another study has shown that the interaction between sleep and immunological processes can be reciprocal, working both ways. After administration of the typhoid vaccine, subjects reported decreased total sleep time and sleep efficiency compared to the placebo group [80]. Authors point out that levels of IL-6 were increased in the study group 2 h after immunization, suggesting that this interleukin is the starting point of the response and may be crucial for the disruptions of sleep continuity. However, correlations between IL-6 and sleep measures were not significant. In this study, blood was taken 2 h after administration of the typhoid vaccine. Thus, the exact level of IL-6 during bedtime is not known. Moreover, IL-6 can probably promote SWS [81].

Sleep emerges as an essential factor in determining vaccine effectiveness, which is associated with immune memory formation. Effects of sleep loss can negatively contribute to antibody levels even one year after the immunization. Importantly, those studies suggest that sleep hygiene should be more stressed, and patients should be properly educated during vaccination appointments.

### 4.2. Immune Memory Formation

It remains elusive which phase of immunological memory formation is influenced by sleep. SWS is proposed to play a role in antigen-presenting cell (APC)-T cells interactions, which are necessary for the formation of immunological memory, as it induces a pro-inflammatory state. In addition, sleep, particularly SWS, decreases the number of APCs cells precursors and T cells in the blood, probably favoring their transport to the lymph nodes, which enhances the chance of their interaction [82] as well as it significantly increases IL-12 levels, but not interferon-α (IFN-α), produced by plasmacytoid dendritic cells [83]. Elevated levels of IL-12 and IL-17 and decreased levels of cortisol create a unique, proinflammatory environment, which promotes the establishment of immunological memory [17]. Hence, SWS is perhaps crucial for the consolidation stage of forming immunological memory, although a decrease of SWS duration is not necessarily associated with habitual sleep loss [17]. Therefore, there may also be other mechanisms through which habitual sleep loss influences the process of immunological memory formation. Authors report a shift in the Th1/Th2 balance toward Th2 dominance in insomnia [84]. This leads to up-regulated response against extracellular organisms, offering a plausible explanation for the lower efficacy of some vaccines in patients with short sleep duration.

During sleep, a unique environment for immune cells occurs, in which IL12 and IFN play a vital role. Possibly, during sleep, interactions between APC-T cells are promoted, therefore sleep, and especially SWS, may be crucial for immune memory formation.

### 4.3. Infectious Diseases Susceptibility

There are also studies investigating the immune responses in patients with sleep deficiency. A study by Orzech et al. [85] used actigraphy to assess the risk of habitual short sleep for developing infectious illnesses in adolescents. Compared to longer sleepers (on average 7 h 28 min, SD = 30 min), the group with short sleep (on average 6 h 24 min, SD = 40 min) was associated with a higher frequency of common illnesses such as cold, flu, and gastroenteritis [85]. Those results are coherent with a previous study by Prather et al., in which participants who slept 6 h or less (sleep was measured by actigraphy) showed higher susceptibility to the common cold [74]. Results were independent of conflicting variables, indicating that sleep may be an independent risk factor for a common cold. Those studies suggest that sleep deficiency can be a favorable factor for the onset of widespread diseases. Primary care physicians should consider putting more emphasis on recommending sleep hygiene to patients during cold and flu seasons.

During the COVID-19 pandemic, there were reports of the associations between sleep loss a week prior to the onset of symptoms and the severity of the disease [75]. Additionally, stress connected to social isolation and unstable financial situations increased the prevalence of sleep disturbances [86]. SARS-CoV-2 enters the cell through the ACE-2 receptor, which is found particularly on the lung type II alveolar cells but also on the cortical neurons and glia, making them susceptible to SARS-CoV-2 invasion [87]. Sleep disturbances were reported in other brain infections [88]. However, it is still unclear whether SARS-CoV-2 could directly induce sleep disturbance, as some patients reported sleepiness or even sleep attacks in the course of COVID-19 [89,90,91], although the cause of these symptoms was not determined.

According to Rodrigues de Silva et al [92]., patients with disrupted circadian rhythms, particularly shift workers, may be more vulnerable to COVID-19 than the general population [92]. Additionally, sleep complaints are one of many post-COVID-19 complications [93,94]. There is still a limited number of evidence regarding the influence of sleep on the efficacy of the COVID-19 vaccines. However, consistent with previous reports, sleep hygiene pre-, and post-vaccine administration needs to be emphasized more

### 4.4. Summary

Proper sleep hygiene significantly improves vaccination efficacy, which is associated with immune memory formation. Crucial phases of this process occur during sleep. Thus, advice on improving sleep quality should be introduced in vaccination centers, particularly for shift workers. Furthermore, sleep loss is also a risk factor for various infections, including viral diseases (Table 3). Therefore, during the COVID-19 pandemic, it is even more critical to accurately diagnose and treat sleep disorders.

## 5. Conclusions

**Figure 2:**
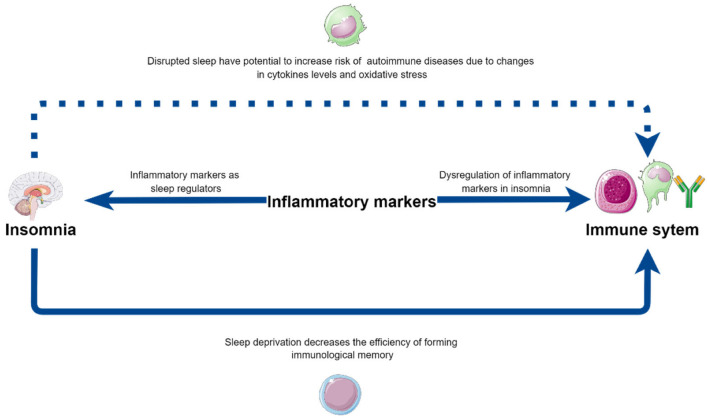
The potential bilateral influence of insomnia on immune system functioning—directly and via inflammatory markers, as well as their contribution to the increased risk of autoimmune diseases in insomniacs. Parts of the figure were drawn by using pictures from Servier Medical Art (http://smart.servier.com/, accessed on 18 June 2022), free and opened software, which is licensed under a Creative Commons Attribution 3.0 Unported License (https://creativecommons.org/licenses/by/3.0/). The picture was designed using diagrams.net website (Diagrams.net version 14.6.13, https://www.diagrams.net/, accessed on 18 June 2022).

Sleep disturbances emerge as a disease influencing not only the central nervous system but also strongly affecting the immune system, with the full spectrum of underlying mechanisms remaining to be unraveled. This group of patients is prone to abnormal immune system function, which may lead to low-grade systemic inflammation, while also increasing the risk of various autoimmune diseases (Table 3). Additionally, sleep medication may have the ability to decrease levels of inflammatory mediators, and monoclonal antibodies may have an impact on sleep quality by targeting the same cytokines. Moreover, the alterations in the interactions between APC and T cells during sleep deprivation need closer investigation, as these alterations could explain the lower vaccination efficacy in those patients (Figure 2). The exact mechanisms of the influence of sleep loss on inflammatory processes are vague, due to the varied methodology of studies in the field, which makes future investigations necessary.

To unify the results, future studies should include the same criteria for diagnosing insomnia, consistent with DSM-V or other widely acknowledged definitions (e.g., ICD-11). This would decrease heterogeneity across the studies on this topic and make their results comparable. A similar effect would be achieved with actigraphy, which may be used to assess the total sleep time, since its use is currently more affordable than PSG. Despite actigraphy limitations, the results of both tools do not differ significantly in average insomnia patients. Additionally, actigraphy would allow for the gathering of data for more than one or two consecutive nights, in contrast to PSG. Subjective methods of insomnia diagnosis should include mainly standardized questionnaires, thereby enabling more accurate comparison between studies.

Sleep disturbances affect not only quality of life but also alter many immune system functions. In everyday practice, this should be taken under consideration in the process of making clinical decisions. Parallel to treating the underlying disease, sleep disturbances should also be treated accordingly, as sleep complaints can worsen the quality of life and the course of other conditions. Current knowledge indicates that appropriate treatment of sleep disorders can lead to decreased frequency of autoimmune disease occurrence and promote host defense.

## Figures and Tables

**Table 1:** Sleep disturbances (with emphasis on insomnia) and alterations of inflammatory markers; a summary of chosen studies.

	J. Fernandez-Mendoza et al. [22]	M.R. Irwin [23]	D.P. Venancio, D. Suchecki [25]	S. Yehuda et al. [26]	I. Burgos et al. [29]
Year	2017	2016	2015	2009	2006
Type of Study	Cohort study	Meta-analysis	Comparative experimental	Comparative experimental	Controlled clinical trial
Patients/Animal Models	adolescents from Penn State Child Cohort	patients	male rats	Male Long Evans hooded rats	Patients
Assessment of Sleep Quality	polysomnography	various	-	-	polysomnography, PSQI, SF-A
Samples/Patients	378	>50,000	34	48	22
Sleep Disturbance	self-reported insomnia symptoms	varied among considered studies	sleep deprivation 18 h/day for 21 days	72 h of REM deprivation	primary insomnia
Results	Adolescents with insomnia symptoms and objective short sleep duration had ↑CRP.	Sleep disturbances associated with ↑CRP and IL-6; insomnia symptoms with ↑IL-6; worse sleep questionnaires scores with ↑IL-6 and CRP; long sleep duration (>8 h) with ↑CRP and IL-6.	↑ plasma TNF-α and IL-6 compared to the control group. ↑ pNFκB in the RPAT of SR21 rats. ↑levels of plasma endotoxin in SR 21 rats.	↑ IL-1α, IL-1β, IL-6, homocysteine, corticosterone, and hyperthermia immediately after 72 h of sleep deprivation. After 7 days of recovery, ↑TNF-α and IL-17A. elevated.	The secretion of IL-6 had a biphasic rhythm in both groups. In insomniacs ↑IL-6 levels during the second half of the night.

Abbreviations: CRP-C-reactive protein IL-6—interleukin 6, IL-10—interleukin 10, IL-1α—interleukin 1 α, IL-1β—interleukin 1 β, IL-17A—interleukin 17A, pIRS-1—plasma insulin receptor substrate 1, pNFκB—plasma nuclear factor kappa-light-chain-enhancer of activated B cells, PSQI—Pittsburgh Sleep Quality Index, REM—rapid eye movement sleep, RPAT—retroperitoneal adipose tissue, SF-A—Schlaffragebogen A, SR21—rats submitted to sleep restriction for 21 days, TLR4—Toll-like receptor 4, TNF-α—tumor necrosis factor.

**Table 2:** Sleep disturbances and risk of immune-mediated diseases.

	YH Hsiao et al. [9]	B. Sivertsen et al. [12]	HM Seo et al. [52]	KA Young et al. [53]
Year	2015	2014	2018	2018
Type of Study	Prospective study	Cohort study	Retrospective cohort analysis	Cohort study
Sample Size	24,715	84,996	154,800	436
Sleep Disturbance	Insomnia	Non-apnea sleep disorder	Diagnosed non-organic sleep disorders (F51) or disorders of initiating and maintaining sleep (G47) according to ICD-10 criteria	Self-reported sleep duration lower than 7 h per night
Results	People afflicted with insomnia are at ↑ risk of developing RA (OR: 1.87, 95% CI 1.29–2.52).	Non-sleep apnea sleep disorders ↑ the risk of SLE (aHR 1.81, 95% CI 1.50–2.18), RA (aHR 1.45, 95% CI 1.36–1.54), AS (aHR 1.53, 95% CI 1.38–1.70), SS (aHR 1.51, 95% CI 1.43–1.60).	Sleep disorders ↑ risk of alopecia areata (OR: 1.913, 95% CI 1.717–2.171), vitiligo (OR: 1.539, 95% CI 1.236–1.917), Graves’ disease (OR: 1.717, 95% CI 1.562–1.886) and Hashimoto disease (OR: 1.641, 95% CI 1.413–1.905).	Relatives of individuals afflicted with SLE who sleep less than 7 h per night have a ↑ risk of developing SLE (aOR 2.8, 95% CI 1.6–5.1).

Abbreviations: aOR—adjusted odds ratio, aHR—adjusted hazard ratio, AS—ankylosing spondylitis, OR—odds ratio, RA—rheumatoid arthritis, SLE—systemic lupus erythematosus, SS—Sjogren syndrome.

**Table 3:** Sleep disturbances and their influence on immune memory formation, vaccine effectiveness, and infectious disease susceptibility.

	S. Taylor et al. [8]	Prather et al. [74]	Sharpley et al. [80]	Orzech et al. [85]	Mello et al. [86]
Year	2017	2015	2016	2014	2020
Type of study	Controlled clinical trial	Controlled clinical trial	Double-blind placebo controlled study	Field-based study	Narrative review
Patients	Young adult college students with Insomnia (65) or No Insomnia (68)	94 men and 70 women aged 18– 55 years	16 healthy male and female adult participants	56 adolescents aged 14–19 years	-
Assessment of Sleep Quality	Questionnaires and self-reported sleep diaries	Actigraphy and self-reported sleep diaries	PSG	Actigraphy and In-Person interviews	-
Measures	HI assay for detecting the presence of anti-influenza antibodies in serum	Viral-specific antibody levels to RV and numerous variables previously associated with susceptibility to the common cold	IL-6 serum levels	Number of illness bouts, illness duration, and absences from school	A thorough investigation of various sleep disturbances treatment and maintenance during the COVID-19 pandemic.
Results	The insomnia group had ↓ baseline antibody levels and remained with ↓ levels four weeks post-vaccination.	Shorter sleep duration was associated with ↑ risk for the development of the cold.	↓ TST and SE% ↑ WASO, total wake, sleep stage transitions, number of awakenings, and awakening index.	A trend for shorter TST in the 6-day window before the illness was found.	-

Abbreviations: HI—Hemagglutination Inhibition, PSG—polysomnography, TST—total sleep time, SE—sleep efficiency, WASO—wake after sleep onset (in minutes), RV—rhinovirus.

## Data Availability

Not applicable.

## References

[1] Bathgate C.J., Edinger J.D., Wyatt J.K., Krystal A.D. (2016). Objective but Not Subjective Short Sleep Duration Associated with Increased Risk for Hypertension in Individuals with Insomnia. Sleep.

[2] Itani O., Jike M., Watanabe N., Kaneita Y. (2017). Short Sleep Duration and Health Outcomes: A Systematic Review, Meta-Analysis, and Meta-Regression. Sleep Med..

[3] AlDabal L., BaHammam A.S. (2011). Metabolic, Endocrine, and Immune Consequences of Sleep Deprivation. Open Respir. Med. J..

[4] Pigeon W.R., Bishop T.M., Krueger K.M. (2017). Insomnia as a Precipitating Factor in New Onset Mental Illness: A Systematic Review of Recent Findings. Curr. Psychiatry Rep..

[5] Katz D.A., McHorney C.A. (1998). Clinical Correlates of Insomnia in Patients With Chronic Illness. Arch. Intern. Med..

[6] Shi L., Chen S.-J., Ma M.-Y., Bao Y.-P., Han Y., Wang Y.-M., Shi J., Vitiello M.V., Lu L. (2018). Sleep Disturbances Increase the Risk of Dementia: A Systematic Review and Meta-Analysis. Sleep Med. Rev..

[7] Irwin M.R., Vitiello M.V. (2019). Implications of Sleep Disturbance and Inflammation for Alzheimer’s Disease Dementia. Lancet Neurol..

[8] Taylor D.J., Kelly K., Kohut M.L., Song K.-S. (2017). Is Insomnia a Risk Factor for Decreased Influenza Vaccine Response?. Behav. Sleep Med..

[9] Hsiao Y.-H., Chen Y.-T., Tseng C.-M., Wu L.-A., Lin W.-C., Su V.Y.-F., Perng D.-W., Chang S.-C., Chen Y.-M., Chen T.-J. (2015). Sleep Disorders and Increased Risk of Autoimmune Diseases in Individuals without Sleep Apnea. Sleep.

[10] Sochal M., Małecka-Panas E., Gabryelska A., Talar-Wojnarowska R., Szmyd B., Krzywdzińska M., Białasiewicz P. (2020). Determinants of Sleep Quality in Inflammatory Bowel Diseases. J. Clin. Med..

[11] Sochal M., Małecka-Panas E., Gabryelska A., Fichna J., Talar-Wojnarowska R., Szmyd B., Białasiewicz P. (2021). Brain-Derived Neurotrophic Factor Is Elevated in the Blood Serum of Crohn’s Disease Patients, but Is Not Influenced by Anti-TNF-α Treatment-A Pilot Study. Neurogastroenterol. Motil..

[12] Sivertsen B., Lallukka T., Salo P., Pallesen S., Hysing M., Krokstad S., Øverland S. (2014). Insomnia as a Risk Factor for Ill Health: Results from the Large Population-Based Prospective HUNT Study in Norway. J. Sleep Res..

[13] Abbas A.K., Lichtman A.H., Pillai S. (2021). Cellular and Molecular Immunology E-Book.

[14] Ordovas-Montanes J., Rakoff-Nahoum S., Huang S., Riol-Blanco L., Barreiro O., Andrian U.H. (2015). von The Regulation of Immunological Processes by Peripheral Neurons in Homeostasis and Disease. Trends Immunol..

[15] Flierl M.A., Rittirsch D., Nadeau B.A., Chen A.J., Sarma J.V., Zetoune F.S., McGuire S.R., List R.P., Day D.E., Hoesel L.M. (2007). Phagocyte-Derived Catecholamines Enhance Acute Inflammatory Injury. Nature.

[16] Pert C.B., Ruff M.R., Weber R.J., Herkenham M. (1985). Neuropeptides and Their Receptors: A Psychosomatic Network. J. Immunol..

[17] Besedovsky L., Lange T., Haack M. (2019). The Sleep-Immune Crosstalk in Health and Disease. Physiological Reviews.

[18] Karatas G., Bal A., Yuceege M., Yalcin E., Firat H., Dulgeroglu D., Karataş F., Sahin S., Cakci A., Ardic S. (2017). The Evaluation of Sleep Quality and Response to Anti-Tumor Necrosis Factor α Therapy in Rheumatoid Arthritis Patients. Clin. Rheumatol..

[19] Zamarrón C., Maceiras F., Mera A., Gómez-Reino J.J. (2004). Effect of the First Infliximab Infusion on Sleep and Alertness in Patients with Active Rheumatoid Arthritis. Ann. Rheum. Dis..

[20] Penner I.-K., Sivertsdotter E.C., Celius E.G., Fuchs S., Schreiber K., Berkö S., Svenningsson A., for the TYNERGY trial investigators (2015). Improvement in Fatigue during Natalizumab Treatment Is Linked to Improvement in Depression and Day-Time Sleepiness. Front. Neurol..

[21] Fan T.-T., Chen W.-H., Shi L., Lin X., Tabarak S., Chen S.-J., Que J.-Y., Bao Y., Tang X.-D., Shi J. (2019). Objective Sleep Duration Is Associated with Cognitive Deficits in Primary Insomnia: BDNF May Play a Role. Sleep.

[22] Fernandez-Mendoza J., Baker J.H., Vgontzas A.N., Gaines J., Liao D., Bixler E.O. (2017). Insomnia Symptoms with Objective Short Sleep Duration Are Associated with Systemic Inflammation in Adolescents. Brain Behav. Immun..

[23] Irwin M.R., Olmstead R., Carroll J.E. (2016). Sleep Disturbance, Sleep Duration, and Inflammation: A Systematic Review and Meta-Analysis of Cohort Studies and Experimental Sleep Deprivation. Biol. Psychiatry.

[24] Ibarra-Coronado E.G., Pantaleón-Martínez A.M., Velazquéz-Moctezuma J., Prospéro-García O., Méndez-Díaz M., Pérez-Tapia M., Pavón L., Morales-Montor J. (2015). The Bidirectional Relationship between Sleep and Immunity against Infections. J. Immunol. Res..

[25] Venancio D.P., Suchecki D. (2015). Prolonged REM Sleep Restriction Induces Metabolic Syndrome-Related Changes: Mediation by pro-Inflammatory Cytokines. Brain Behav. Immun..

[26] Yehuda S., Sredni B., Carasso R.L., Kenigsbuch-Sredni D. (2009). REM Sleep Deprivation in Rats Results in Inflammation and Interleukin-17 Elevation. J. Interferon. Cytokine Res..

[27] Baracchi F., Opp M.R. (2008). Sleep-Wake Behavior and Responses to Sleep Deprivation of Mice Lacking Both Interleukin-1 Beta Receptor 1 and Tumor Necrosis Factor-Alpha Receptor 1. Brain Behav. Immun..

[28] Deboer T., Fontana A., Tobler I. (2002). Tumor Necrosis Factor (TNF) Ligand and TNF Receptor Deficiency Affects Sleep and the Sleep EEG. J. Neurophysiol..

[29] Burgos I., Richter L., Klein T., Fiebich B., Feige B., Lieb K., Voderholzer U., Riemann D. (2006). Increased Nocturnal Interleukin-6 Excretion in Patients with Primary Insomnia: A Pilot Study. Brain Behav. Immun..

[30] Carroll J.E., Carrillo C., Olmstead R., Witarama T., Breen E.C., Yokomizo M., Seeman T., Irwin M.R. (2015). Sleep Deprivation and Divergent Toll-like Receptor-4 Activation of Cellular Inflammation in Aging. Sleep.

[31] Jewett K.A., Krueger J.M. (2012). Humoral Sleep Regulation; Interleukin-1 and Tumor Necrosis Factor. Vitam. Horm..

[32] Krueger J.M., Clinton J.M., Winters B.D., Zielinski M.R., Taishi P., Jewett K.A., Davis C.J. (2011). Involvement of Cytokines in Slow Wave Sleep. Prog. Brain Res..

[33] Chung K.-F., Yeung W.-F., Ho F.Y.-Y., Yung K.-P., Yu Y.-M., Kwok C.-W. (2015). Cross-Cultural and Comparative Epidemiology of Insomnia: The Diagnostic and Statistical Manual (DSM), International Classification of Diseases (ICD) and International Classification of Sleep Disorders (ICSD). Sleep Med..

[34] Slavish D.C., Graham-Engeland J.E., Engeland C.G., Taylor D.J., Buxton O.M. (2018). Insomnia Symptoms Are Associated with Elevated C-Reactive Protein in Young Adults. Psychol. Health.

[35] Prather A.A., Vogelzangs N., Penninx B.W.J.H. (2015). Sleep Duration, Insomnia, and Markers of Systemic Inflammation: Results from the Netherlands Study of Depression and Anxiety (NESDA). J. Psychiatr. Res..

[36] Vgontzas A.N., Fernandez-Mendoza J., Liao D., Bixler E.O. (2013). Insomnia with Objective Short Sleep Duration: The Most Biologically Severe Phenotype of the Disorder. Sleep Med. Rev..

[37] Watson N.F., Buchwald D., Delrow J.J., Altemeier W.A., Vitiello M.V., Pack A.I., Bamshad M., Noonan C., Gharib S.A. (2017). Transcriptional Signatures of Sleep Duration Discordance in Monozygotic Twins. Sleep.

[38] Jaehne E.J., Corrigan F., Toben C., Jawahar M.C., Baune B.T. (2015). The Effect of the Antipsychotic Drug Quetiapine and Its Metabolite Norquetiapine on Acute Inflammation, Memory and Anhedonia. Pharmacol. Biochem. Behav..

[39] Turra B.O., Barbisan F., Azzolin V.F., Teixeira C.F., Flores T., Braun L.E., de Oliveira Nerys D.A., Rissi V.B., de Oliveira Alves A., Assmann C.E. (2020). Unmetabolized Quetiapine Exerts an in Vitro Effect on Innate Immune Cells by Modulating Inflammatory Response and Neutrophil Extracellular Trap Formation. Biomed Pharm..

[40] Daniele S., Da Pozzo E., Zappelli E., Martini C. (2015). Trazodone Treatment Protects Neuronal-like Cells from Inflammatory Insult by Inhibiting NF-ΚB, P38 and JNK. Cell. Signal..

[41] Tarocco A., Caroccia N., Morciano G., Wieckowski M.R., Ancora G., Garani G., Pinton P. (2019). Melatonin as a Master Regulator of Cell Death and Inflammation: Molecular Mechanisms and Clinical Implications for Newborn Care. Cell Death Dis..

[42] Hardeland R. (2018). Melatonin and Inflammation—Story of a Double-Edged Blade. J. Pineal Res..

[43] Rockstrom M.D., Chen L., Taishi P., Nguyen J.T., Gibbons C.M., Veasey S.C., Krueger J.M. (2018). Tumor Necrosis Factor Alpha in Sleep Regulation. Sleep Med. Rev..

[44] Weinberger J.F., Raison C.L., Rye D.B., Montague A.R., Woolwine B.J., Felger J.C., Haroon E., Miller A.H. (2015). Inhibition of Tumor Necrosis Factor Improves Sleep Continuity in Patients with Treatment Resistant Depression and High Inflammation. Brain Behav. Immun..

[45] Wolfe F., Michaud K., Li T. (2006). Sleep Disturbance in Patients with Rheumatoid Arthritis: Evaluation by Medical Outcomes Study and Visual Analog Sleep Scales. J. Rheumatol..

[46] Karatas G., Bal A., Yuceege M., Firat H., Gurcay E., Ardic S., Cakci F.A. (2018). Evaluation of Sleep Quality in Patients with Ankylosing Spondylitis and Efficacy of Anti-TNF-α Therapy on Sleep Problems: A Polisomnographic Study. Int. J. Rheum. Dis..

[47] Fragiadaki K., Tektonidou M.G., Konsta M., Chrousos G.P., Sfikakis P.P. (2012). Sleep Disturbances and Interleukin 6 Receptor Inhibition in Rheumatoid Arthritis. J. Rheumatol..

[48] Savard J., Laroche L., Simard S., Ivers H., Morin C.M. (2003). Chronic Insomnia and Immune Functioning. Psychosom. Med..

[49] Lange T., Dimitrov S., Fehm H.-L., Born J. (2006). Sleep-like Concentrations of Growth Hormone and Cortisol Modulate Type1 and Type2 in-Vitro Cytokine Production in Human T Cells. Int. Immunopharmacol..

[50] Fondell E., Axelsson J., Franck K., Ploner A., Lekander M., Bälter K., Gaines H. (2011). Short Natural Sleep Is Associated with Higher T Cell and Lower NK Cell Activities. Brain Behav. Immun..

[51] Lasselin J., Rehman J., Åkerstedt T., Lekander M., Axelsson J. (2015). Effect of Long-Term Sleep Restriction and Subsequent Recovery Sleep on the Diurnal Rhythms of White Blood Cell Subpopulations. Brain Behav. Immun..

[52] Seo H.-M., Kim T.L., Kim J.S. (2018). The Risk of Alopecia Areata and Other Related Autoimmune Diseases in Patients with Sleep Disorders: A Korean Population-Based Retrospective Cohort Study. Sleep.

[53] Young K.A., Munroe M.E., Harley J.B., Guthridge J.M., Kamen D.L., Gilkensen G.S., Weisman M.H., Karp D.R., Wallace D.J., James J.A. (2018). Less than 7 Hours of Sleep per Night Is Associated with Transitioning to Systemic Lupus Erythematosus. Lupus.

[54] Palma B.D., Gabriel A., Colugnati F.A.B., Tufik S. (2006). Effects of Sleep Deprivation on the Development of Autoimmune Disease in an Experimental Model of Systemic Lupus Erythematosus. Am. J. Physiol. Regul. Integr. Comp. Physiol..

[55] Sahraian M.A., Rezaali S., Hosseiny M., Doosti R., Tajik A., Naser Moghadasi A. (2017). Sleep Disorder as a Triggering Factor for Relapse in Multiple Sclerosis. Eur. Neurol..

[56] Caloz E., Vullièmoz M., Haba Rubio J., Pittet V., Mamadou Barry P., Michetti P., Heinzer R., Maillard M.H. (2020). P808 Prevalence and Factors Associated with Sleep Disturbances in Inflammatory Bowel Disease Patients Compared with Normal Controls. J. Crohn’s Colitis.

[57] kotb H.A., Rady H.M., Ghanim D.H. (2013). Sleep Disturbance in Female Patients with Systemic Lupus Erythematosus and Its Relation to Disease Parameters. Egypt. Rheumatol..

[58] Guo G., Fu T., Yin R., Zhang L., Zhang Q., Xia Y., Li L., Gu Z. (2016). Sleep Quality in Chinese Patients with Rheumatoid Arthritis: Contributing Factors and Effects on Health-Related Quality of Life. Health Qual. Life Outcomes.

[59] Saçmacı H., Gürel G. (2019). Sleep Disorders in Patients with Psoriasis: A Cross-Sectional Study Using Non-Polysomnographical Methods. Sleep Breath.

[60] Boeschoten R.E., Braamse A.M.J., Beekman A.T.F., Cuijpers P., van Oppen P., Dekker J., Uitdehaag B.M.J. (2017). Prevalence of Depression and Anxiety in Multiple Sclerosis: A Systematic Review and Meta-Analysis. J. Neurol. Sci..

[61] Viana P., Rodrigues E., Fernandes C., Matas A., Barreto R., Mendonça M., Peralta R., Geraldes R. (2015). InMS: Chronic Insomnia Disorder in Multiple Sclerosis-a Portuguese Multicentre Study on Prevalence, Subtypes, Associated Factors and Impact on Quality of Life. Mult. Scler. Relat. Disord..

[62] Bamer A., Johnson K., Amtmann D., Kraft G. (2008). Prevalence of Sleep Problems in Individuals with Multiple Sclerosis. Mult. Scler..

[63] Ditmer M., Gabryelska A., Turkiewicz S., Białasiewicz P., Małecka-Wojciesko E., Sochal M. (2022). Sleep Problems in Chronic Inflammatory Diseases: Prevalence, Treatment, and New Perspectives: A Narrative Review. J. Clin. Med..

[64] Kinnucan J.A., Rubin D.T., Ali T. (2013). Sleep and Inflammatory Bowel Disease: Exploring the Relationship Between Sleep Disturbances and Inflammation. Gastroenterol. Hepatol..

[65] Vaccaro A., Dor Y.K., Nambara K., Pollina E.A., Lin C., Greenberg M.E., Rogulja D. (2020). Sleep Loss Can Cause Death through Accumulation of Reactive Oxygen Species in the Gut. Cell.

[66] Scavuzzi B.M., Simão A.N.C., Iriyoda T.M.V., Lozovoy M.A.B., Stadtlober N.P., Franchi Santos L.F.D.R., Flauzino T., de Medeiros F.A., de Sá M.C., Consentin L. (2018). Increased Lipid and Protein Oxidation and Lowered Anti-Oxidant Defenses in Systemic Lupus Erythematosus Are Associated with Severity of Illness, Autoimmunity, Increased Adhesion Molecules, and Th1 and Th17 Immune Shift. Immunol. Res..

[67] Teixeira K.R.C., dos Santos C.P., de Medeiros L.A., Mendes J.A., Cunha T.M., De Angelis K., Penha-Silva N., de Oliveira E.P., Crispim C.A. (2019). Night Workers Have Lower Levels of Antioxidant Defenses and Higher Levels of Oxidative Stress Damage When Compared to Day Workers. Sci. Rep..

[68] Bertolotti M., Yim S.H., Garcia-Manteiga J.M., Masciarelli S., Kim Y.-J., Kang M.-H., Iuchi Y., Fujii J., Vené R., Rubartelli A. (2010). B- to Plasma-Cell Terminal Differentiation Entails Oxidative Stress and Profound Reshaping of the Antioxidant Responses. Antioxid. Redox Signal..

[69] Hurtado-Alvarado G., Pavón L., Castillo-García S.A., Hernández M.E., Domínguez-Salazar E., Velázquez-Moctezuma J., Gómez-González B. (2013). Sleep Loss as a Factor to Induce Cellular and Molecular Inflammatory Variations. Clin. Dev. Immunol..

[70] Voderholzer U., Fiebich B.L., Dersch R., Feige B., Piosczyk H., Kopasz M., Riemann D., Lieb K. (2012). Effects of Sleep Deprivation on Nocturnal Cytokine Concentrations in Depressed Patients and Healthy Control Subjects. JNP.

[71] Ananthakrishnan A.N., Long M.D., Martin C.F., Sandler R.S., Kappelman M.D. (2013). Sleep Disturbance and Risk of Active Disease in Patients with Crohn’s Disease and Ulcerative Colitis. Clin. Gastroenterol. Hepatol..

[72] Prather A.A., Hall M., Fury J.M., Ross D.C., Muldoon M.F., Cohen S., Marsland A.L. (2012). Sleep and Antibody Response to Hepatitis B Vaccination. Sleep.

[73] Everson C.A. (1993). Sustained Sleep Deprivation Impairs Host Defense. Am. J. Physiol. -Regul. Integr. Comp. Physiol..

[74] Prather A.A., Janicki-Deverts D., Hall M.H., Cohen S. (2015). Behaviorally Assessed Sleep and Susceptibility to the Common Cold. Sleep.

[75] Huang B., Niu Y., Zhao W., Bao P., Li D. (2020). Reduced Sleep in the Week Prior to Diagnosis of COVID-19 Is Associated with the Severity of COVID-19. Nat. Sci. Sleep.

[76] Lange T., Perras B., Fehm H.L., Born J. (2003). Sleep Enhances the Human Antibody Response to Hepatitis A Vaccination. Psychosom. Med..

[77] Lange T., Born J., Westermann J. (2019). Sleep Matters: CD4+ T Cell Memory Formation and the Central Nervous System. Trends Immunol..

[78] CDC-Data and Statistics-Sleep and Sleep Disorders. https://www.cdc.gov/sleep/data_statistics.html.

[79] Prather A.A., Pressman S.D., Miller G.E., Cohen S. (2021). Temporal Links Between Self-Reported Sleep and Antibody Responses to the Influenza Vaccine. Int. J. Behav. Med..

[80] Sharpley A.L., Cooper C.M., Williams C., Godlewska B.R., Cowen P.J. (2016). Effects of Typhoid Vaccine on Inflammation and Sleep in Healthy Participants: A Double-Blind, Placebo-Controlled, Crossover Study. Psychopharmacology.

[81] Benedict C., Scheller J., Rose-John S., Born J., Marshall L. (2009). Enhancing Influence of Intranasal Interleukin-6 on Slow-Wave Activity and Memory Consolidation during Sleep. FASEB J..

[82] Born J., Lange T., Hansen K., Mölle M., Fehm H.L. (1997). Effects of Sleep and Circadian Rhythm on Human Circulating Immune Cells. J. Immunol..

[83] Dimitrov S., Lange T., Nohroudi K., Born J. (2007). Number and Function of Circulating Human Antigen Presenting Cells Regulated by Sleep. Sleep.

[84] Sakami S., Ishikawa T., Kawakami N., Haratani T., Fukui A., Kobayashi F., Fujita O., Araki S., Kawamura N. (2002). Coemergence of Insomnia and a Shift in the Th1/Th2 Balance toward Th2 Dominance. NIM.

[85] Orzech K.M., Acebo C., Seifer R., Barker D., Carskadon M.A. (2014). Sleep Patterns Are Associated with Common Illness in Adolescents. J. Sleep Res..

[86] Mello M.T.D., Silva A., Guerreiro R.D.C., da-Silva F.R., Esteves A.M., Poyares D., Piovezan R., Treptow E., Starling M., Rosa D.S. (2020). Sleep and COVID-19: Considerations about Immunity, Pathophysiology, and Treatment. Sleep Sci..

[87] Crook H., Raza S., Nowell J., Young M., Edison P. (2021). Long COVID—Mechanisms, Risk Factors, and Management. BMJ.

[88] Tesoriero C., Del Gallo F., Bentivoglio M. (2019). Sleep and Brain Infections. Brain Res. Bull..

[89] Najafi A., Sadeghniiat-Haghighi K., Alemohammad Z.B., Akbarpour S. (2020). COVID-19: Sleep Research Perspectives. Sleep Sci..

[90] Szmyd B., Bartoszek A., Karuga F.F., Staniecka K., Błaszczyk M., Radek M. (2021). Medical Students and SARS-CoV-2 Vaccination: Attitude and Behaviors. Vaccines.

[91] Szmyd B., Karuga F.F., Bartoszek A., Staniecka K., Siwecka N., Bartoszek A., Błaszczyk M., Radek M. (2021). Attitude and Behaviors towards SARS-CoV-2 Vaccination among Healthcare Workers: A Cross-Sectional Study from Poland. Vaccines.

[92] Silva F.R.D., Guerreiro R.D.C., Andrade H.D.A., Stieler E., Silva A., de Mello M.T. (2020). Does the Compromised Sleep and Circadian Disruption of Night and Shiftworkers Make Them Highly Vulnerable to 2019 Coronavirus Disease (COVID-19)?. Chronobiol. Int..

[93] Huang C., Huang L., Wang Y., Li X., Ren L., Gu X., Kang L., Guo L., Liu M., Zhou X. (2021). 6-Month Consequences of COVID-19 in Patients Discharged from Hospital: A Cohort Study. Lancet.

[94] Carod-Artal F.J. (2021). Post-COVID-19 Syndrome: Epidemiology, Diagnostic Criteria and Pathogenic Mechanisms Involved. Rev. Neurol..

